# Prevalence and Influencing Factors of Overweight and Obesity among Adult Residents of Western China: A Cross-Sectional Study

**DOI:** 10.1155/2021/9919443

**Published:** 2021-10-13

**Authors:** Li Zheng, Feng Deng, Honglin Wang, Biao Yang, Meng Qu, Peirong Yang

**Affiliations:** Baoji Center for Disease Control and Prevention, Baoji 721000, China

## Abstract

**Background:**

Overweight and obesity have become a serious health problem. There are a few data on the prevalence of overweight and obesity in Baoji city of western China, this study was conducted to investigate the epidemiologic features of overweight and obesity and explored influencing factors among Baoji adult residents.

**Methods:**

A cross-sectional study, including 36,600 participants aged above 15 years, was carried out in Baoji city in 2018. Each participant's weight and height were measured, and demographic and behavioral characteristics were collected using questionnaires. Data were analyzed by means of logistic regression considering 95% level of significance.

**Results:**

Overall, the prevalence of overweight and obesity was 30.73% and 3.11%, respectively. Male had a significantly higher prevalence of overweight (31.45% vs. 29.98%, *P* < 0.05) while female had a higher prevalence of obesity (3.50 vs. 2.74, *P* < 0.001). In the logistic regression analysis, being married or living with a partner (OR = 1.266, *P* < 0.001), unemployed or retired (OR = 1.183, *P* < 0.001), former smokers (OR = 1.116, *P* < 0.05), drinking alcohol (OR = 1.410, *P* < 0.001), sleeping more than 10 hours (OR = 1.274, *P* < 0.001), and increasing age were all significantly associated with a higher prevalence of overweight/obesity, whereas people who lived in rural areas (*R* = 0.904, *P* < 0.001) or had a sufficient leisure time physical activity per week (*R* = 0.945, *P* < 0.05) were associated with a lower prevalence.

**Conclusion:**

Our results demonstrate that demographic and behavioral factors play an important role in prevalence of overweight/obesity, which can support the implementation of interventions aimed at weight control and consequently prevention of related diseases in this population.

## 1. Introduction

Obesity has become a major public health problem; according to a recent report by the WHO, more than 1.9 billion adults were overweight, and of these over 650 million were obese, the worldwide prevalence of obesity nearly tripled between 1975 and 2016 [1]. The effect of overweight and obesity on health has been well documented in the literature; they are major risk factors for a number of chronic diseases, including diabetes, hypertension, musculoskeletal disorders, cardiovascular diseases, and some types of cancer [2].

Researchers found the prevalence of overweight and obesity varied across countries in the levels and trends with distinct regional patterns, and there were likely to be continued increases of obesity epidemic in developing countries [3]. As is known to all, China is the largest developing country, with a large economic development over the past several decades. Its rapid economic growth has provoked many changes in lifestyles involving dietary habits and physical activity, which have contributed to an increase in body weight [4]. Recent studies suggest that the prevalence of overweight and obesity in the Chinese population increased from 37.4% to 41.2% and 8.6% to 12.9% between 2000 and 2014, with an estimated increase of 0.27% and 0.32% per year, respectively [5]. Hence, prevention and control of overweight and obesity are of great urgency in China.

The rise in overweight and obesity arouse wide public concern and has led to widespread calls for regular monitoring of changes in overweight and obesity prevalence in all populations. The Chinese government has realized the importance of obesity control and issued Health China Action (2019-2030), which lists reducing the growth rate of obesity as one of its key tasks. Baoji city is located in western China, the less developed areas. To date, this is the first large representative population-based survey of chronic diseases in this area; the aims of this study were to provide the recent estimates of the prevalence of overweight and obesity and to explore potential influencing factors in western China, which will useful for policy makers in formulating policies for obesity management.

## 2. Methods

### 2.1. Subjects

Data for the present study was from the Prevalence of Major Chronic Diseases and Related Risk Factors Survey in Baoji city, western China. This was a population-based cross-sectional survey; we used a multistage cluster sampling method to select a representative sample of people aged above 15 years. The first stage covered all twelve districts and counties in Baoji city. Second, five streets (for urban areas) or towns (for rural areas) were selected from each district or county using the probability-proportional-to-size (PPS) method. Third, four neighbourhood committees (for urban areas) or villages (for rural areas) were further randomly selected from each street (for urban areas) or town (for rural areas), also using the PPS method. Fourth, in each neighbourhood committees (for urban areas) or village (for rural areas), fifty-sixty households were randomly chosen using the simple random sampling method. In the final stage, one person aged above 15 years, who was a local registered resident for more than 6 months was selected randomly from each chosen household using a Kish selection table. The ultimate target sample size was established to be 37,000, while 400 participants with missing information such as gender, age, weight, and height were excluded; therefore, 36,600 participants were included in the analysis, accounting for about 11‰ of the total adult population of Baoji city. This work was approved by Baoji Center for Disease Control and Prevention Academic Ethics Board. Written informed consent was acquired from each participant in the survey.

### 2.2. Data Collection

Participants were required to complete a questionnaire to collect demographic characteristics (including age, gender, location, marital status, education, and occupation) and behavioral characteristics (including smoking, drinking, sleep duration, and physical activity) through face-to-face interviews by trained medical staff. Body height and weight were measured without shoes, and in light clothing after overnight fasting, height was measured to the nearest 0.1 cm, and weight was measured to the nearest 0.1 kg.

### 2.3. Definition of Variables

Overweight and obesity were defined as a BMI of 24–27.9 kg/m^2^ and a BMI ≥ 28 kg/m^2^, respectively, by the Chinese standards [6]. Education was classified into three levels: primary school and lower (receiving only primary education or no education); middle school (including junior middle school, senior middle school, and secondary vocational schooling); and college and higher. The occupations consisted of three parts: manual worker, nonmanual worker, and unemployed and retired people. Smoking status was categorized into: current, former, or never smoker [7]. A drinker was defined as a person who had consumed more than one alcoholic drink a week during the previous year, including any form of alcohol [8]. Sleep duration was classified into four levels: <6 h, 6-8 h, 8-10 h, and >10 h; “<6 h” was defined as persons who slept less than 6 hours over 3 days a week, and those who slept more than 10 hours over 3 days a week were defined as “>10 h” [9]. Physical activity recommendations of the WHO for health were considered to be satisfied if participants reported engaging in at least 150 minutes of moderate-intensity aerobic physical activity or 75 minutes of vigorous intensity activity throughout the week or an equivalent combination of moderate and vigorous intensity activity [10]. To assess leisure time physical activity, participants were asked to report the number of exercise days in a usual week, duration of exercise per day, and exercise intensity. We classified leisure time physical activity level in this study as sufficient versus insufficient.

### 2.4. Statistical Analysis

Categorical variables were described as numbers and percentages. Continuous variables were presented as the mean ± standard derivation (SD). Between-group differences in participant characteristics were tested using a *t*-test for continuous variables and chi-square/Cochran-Mantel-Haenszel test for categorical variables. Separate univariate analyses were used to identify those variables associated with overweight/obesity among subjects in the populations studied. All these significant variables were included in the multivariable logistic regression models. Results of logistic regression analysis were presented together with OR and 95% confidence intervals (CI). All statistical analyses were conducted using IBM SPSS Statistics Version 19.0, and a *P* ≤ 0.05 was considered statistically significant.

## 3. Results

### 3.1. Basic Characteristics

The basic characteristics of the study participants are illustrated in Table 1. A total of 36,600 participants were included in the analysis, including 18,676 (51.03%) males and 17,924 (48.97%) females and 52.33% lived in urban areas and 47.67% lived in rural areas. The mean age of 41.61 ± 16.32 years. More than three quarters (75.37%) of the residents were married or living with a partner, 60.43% had an education level of middle school, and 73.62% were manual worker. Current smokers and alcohol drinkers accounted for 17.46% and 5.84%. About 31.18% of participants had a sufficient leisure time physical activity. Compared with females, males showed higher values for height, weight, and BMI.

### 3.2. Prevalence of Overweight and Obesity

The overall prevalence of overweight and obesity was 30.73% and 3.11% in Baoji (Table 2). In males, 31.45% were overweight and 2.74% were obesity. Likewise, 29.98% were overweight and 3.50% were obesity in females. According to the WHO BMI classification, the prevalence of overweight (BMI 25–29.9 kg/m^2^) and obesity (BMI ≥ 30 kg/m^2^) was 20.43% (19.51% in male, 21.40% in female) and 0.78% (0.67% in male, 0.90% in female), respectively.

**Figure 1:**
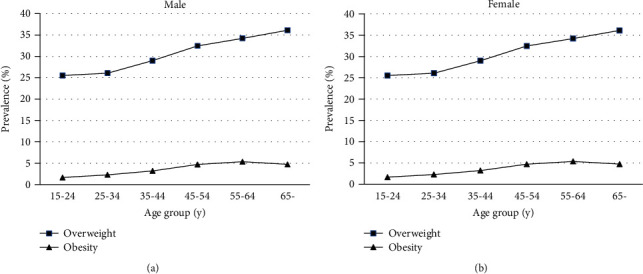
Prevalence of overweight and obesity by different age groups in (a) males and (b) females.

As shown in Figure 1, the prevalence of overweight for male increased with age, peaking at 55~64 years (37.25%); such age trend was not seen in the prevalence of obesity. For female, a gradual increase was noted in the prevalence of overweight from the youngest age group (25.53%) up to the group aged above 65 years (36.11%), while the prevalence of obesity reached its peak at the group aged 55~64 years (5.35%) and then followed by a decline in the higher age group. Moreover, the prevalence of overweight was higher in male than in female in the group aged over 25 years, and the prevalence of obesity was higher in female than in male in the age group 15~24 and aged above 45 years.

The prevalence of overweight and obesity was higher (33.06%, 3.63%) among participants who were married or living with a partner than people who were separated, divorced, or widowed (31.84%, 2.94%) or who were single (22.31%, 1.30%). The higher prevalence of overweight or obesity was found in unemployed and retired people (35.54%, 3.63%). A lower educational level was also associated with higher prevalence of overweight and obesity. As with people residing in rural areas, participants who have a sufficient leisure time physical activity also had a lower prevalence of overweight/obesity. Overweight/obesity was also more prevalent among former smokers and drinkers. In addition, prevalence of overweight was higher (35.86%) in participants who had a ≥10-hour sleep duration, while the highest prevalence of obesity (5.86%) was found in participants with <6-hour sleep duration.

### 3.3. Related Factors for Overweight/Obesity

All significant factors in Table 2 added to the multivariate logistic regression model to assess the significant determinants of combined overweight and obesity (overweight/obesity), as seen in Table 3. Compared with participants aged 15-24 years, people aged 35-44 years, 45-54 years, 55-64 years, and ≥65 years had a greater correlation with developing overweight and obesity (all *P* < 0.001). Overweight/obesity seems to increase for participants who have been married or living with a partner (OR = 1.266, *P* < 0.001), unemployed and retired people (OR = 1.183, *P* < 0.001), former smokers (OR = 1.116, *P* < 0.05), drinking alcohol (OR = 1.410, *P* < 0.001), and those sleeping more than 10 hours (OR = 1.274, *P* < 0.001) and decrease with people who lived in rural areas and had a sufficient leisure time physical activity (*R* = 0904, *P* < 0.001; *R* = 0.945, *P* < 0.05).

## 4. Discussion

Baoji is located in western China with a low level of economic development; there are few data and studies on overweight and obesity. The present study represents the first attempt to examine the prevalence and explore related factors of overweight and obesity in Baoji, using the most recent data from a large representative sample.

The prevalence of overweight and obesity was 30.73% and 3.11% in the analyzed population in Baoji; standardized prevalence by Chinese census data was 30.66% and 3.08%. The prevalence of overweight was comparable to the national level of 30.1%, while obesity was lower than the national level of 11.9% [11]. The figures reported from other regions conducted in China have varied considerably: a study in Jilin province of northeast China in 2012 indicated that the prevalence of overweight and obesity was 32.3% and 14.6% [12]; figures in Zhejiang province of eastern China in 2012 were 32.0% and 6.7% [13], while the prevalence was 25.8% and 7.9% in 2014 in Jiangxi province located in eastern China [14]. It was noted that the prevalence of obesity observed in our study population is considerably lower than the values reported in other regions, but comparable to that of Hanzhong city, northwest China [15]. Differences in the prevalence of overweight and obesity may be due to economic development level, sociodemographic variables, climate or lifestyle habits, and economic development levels [16].

Our study indicated that male has a slightly higher prevalence of overweight than female (31.45% vs. 29.98%), while a negative association was found with obesity (2.74% vs. 3.50), as found in a study including 31 countries' samples based on the International Social Survey Program (38.2% vs. 25.5%, 12.1% vs. 12.5%) [17]. However, in the multivariate analysis, it showed there was no significant gender difference of combined overweight and obesity. Yet, most literatures have found the prevalence of obesity in adults was greater for female than for male, and researchers have also identified biological, behavioral, and socioeconomic factors contributing to the differences in obesity prevalence between genders [18].

Our study found overweight/obesity was more common in urban than in rural areas (35.03% vs. 32.54%), as found in other studies [19]. Along with economic growth and the urbanization of lifestyle in China, the prevalence of overweight/obesity among both urban and rural residents has been on the rise; of particular significance, the disparity between them is narrowing. Studies from four large-scale surveys in China between 2000 and 2014 found the prevalence of overweight in the general Chinese population was 38.9% for urban residents and 34.3% for rural residents in 2000; by 2014, the prevalence was 41.4% for urban residents and 40.7% for rural residents [5]. For a large, but economically unevenly developed country like China, an appreciation of the regional distribution of obesity would be informative with respect to understanding the obesity-related health issues. The long-term trends in overweight/obesity for urban and rural residents in western China, the less developed areas, have not been characterised clearly, and it needs to be studied in the future.

It is important to note that ageing also contributes to a higher prevalence of overweight and obesity; the general prevalence reached its peak at group aged above 65 years and 55~64 years, which is consistent with a study in Beijing [20]. It also should be noted that the prevalence of obesity was higher at a younger age (35~44 years) for male, which may be related to the increasing work pressure, decreased physical activity, and unhealthy lifestyle. Another emerging challenge that should not be ignored is the change in food shopping habits induced by the fast growing online-to-offline food delivery service in China, which may decrease the amount of individual physical activity by keeping people at worksite or home and likely raise the risk of being overweight or obesity [21, 22]. Interestingly, we found the prevalence of obesity was higher in female aged above 45 years than in male, aside from the hormone changes during menopausal transition that also could be partly explained by the fact that young women paid more attention to their body weight and had a greater pressure to keep a slim figure [23]. These data implied that the overweight and obesity management in Baoji area should post 35 to 44-year-old men and postmenopausal women as the priority management groups. Multivariate analysis showed that people who were married or living with a partner were more likely to be overweight/obesity than unmarried people. Previous researches suggested that marriage was associated with weight gain and that divorce and widowhood were associated with weight loss [24, 25]. While a review indicated that marital transitions are more important than marital status in predicting change in body weight, it was found that transition into marriage appears to be associated with weight gain, whereas transition out of marriage is associated with weight loss [26]. Further research is needed to illustrate the relationships between marital transitions and body weight in our study area.

The relationship between smoking and obesity is complex, and published studies have produced conflicting results. Some reported current smokers were less likely to be obese than never smokers [27]; some have shown no significant association between smoking and body mass index [28]; our study is consistent with studies that have found that former smokers were more likely to be obese [29]. These contradictory findings show that the association still requires more attention. Nowadays, recreational alcohol intake is common across the globe, and numerous studies have examined the effect of alcohol intake on obesity. Some previous studies showed no association or a negative association between alcohol intake and weight gain or obesity [30]. In contrast, we found a positive impact of drinking alcohol on overweight/obesity in line with some literatures [31, 32]. Furthermore, other studies have declared that only excessive or heavy drinking is correlated with increased body weight [33]. Apparently, available evidence on the topic is mixed and conflicting, which warrant further exploration.

Our study found a positive association of long sleep duration (>10 h) on overweight/obesity, in accordance with previous studies [34]. Yet, numerous studies showed a significant increased risk of weight gain with inadequate sleep [35]. Further studies are required to confirm such an association. Physical activity contributes to energy expenditure, prevents obesity, and reduces the risk of major NCDs and decreased all-cause mortality [36]. We found there was a lower incidence of overweight/obesity in participants with a sufficient leisure time physical activity, which is consistent with previous studies [37], while it differs from a study which reported that physical inactivity was inversely associated with overweight but not associated with obesity [17]. Moreover, some found there was no significant correlation between regular physical activity and a lower risk of overweight/obesity [19]. Such findings are probably explained by the difference in criteria used to classify participants as physically active or inactive. Our assessment of physical activity focused on leisure time domain and did not include physical activity in transportation, occupational, and household domains, which are major contributors to total physical activity. Thus, further studies are needed to elucidate the potential effect of inclusion of other types of physical activity. The Chinese government has realized the importance of obesity control and, therefore, has implemented several measures to promote participation in physical activity; figures showed these initiatives have already had some effect [5]. Nevertheless, continued nationwide or local interventions are still needed for promotion of physical activity and other healthy lifestyles.

Several limitations should be noted. First, our study was a cross-sectional design, and therefore, causal relations cannot be established. Second, dietary habits could impact on body weight or obesity, and difference in eating habits may be the main reasons for variations in overweight and obesity prevalence across regions. However, we only investigated a few defined major risk factors. In order to better control overweight and obesity, further research is warranted that includes broader factors. Third, as the study is confined to western China, the conclusions from our study cannot represent the situation in other areas in China. Despite these limitations, this is the first study to determine the prevalence of overweight and obesity and explore related factors based on a relatively large sample size in Baoji, which provides important clues for the prevention and control of local obesity.

## 5. Conclusions

In summary, the prevalence of overweight is almost equivalent to that of China and the prevalence of obesity is relatively lower in Baoji district. Factors significantly associated with an increased risk of overweight/obesity were older age, married condition, unemployed or retired, former smoker, drinking alcohol, and sleeping too much, while living in rural areas or having a sufficient physical activity may be a protect factor. This study provides data on current overweight and obesity prevalence and related factors in Baoji city of western China, which may guide the development of practical and effective strategies for managing and preventing overweight and obesity.

## Figures and Tables

**Table 1:** Descriptive characteristics of participants in Baoji, western China.

Characteristic	Male	Female	Total
Number, *n* (%)	18676 (51.03)	17924 (48.97)	36600 (100)
Age (years), mean (SD)	41.00 ± 16.36	42.24 ± 16.26	41.61 ± 16.32
Location, *n* (%)^∗^			
Urban	9595 (51.38)	9557 (53.32)	19152 (52.33)
Rural	9081 (48.62)	8367 (46.68)	17558 (47.67)
Marriage, *n* (%)^∗^			
Single	4646 (24.88)	3139 (17.51)	7785 (21.27)
Married or living with a partner	13464 (72.09)	14120 (78.78)	27584 (75.37)
Separated, divorced, or widowed	566 (3.03)	665 (3.71)	1231 (3.36)
Education, *n* (%)^∗^			
Primary school and lower	3879 (20.77)	5864 (32.72)	9743 (26.62)
Middle school	12222 (65.44)	9897 (55.22)	22119 (60.43)
College and higher	2575 (13.79)	2163 (12.07)	4738 (12.95)
Occupation, *n* (%)^∗^			
Manual worker	13968 (74.79)	12978 (72.41)	26946 (73.62)
Nonmanual worker	3350 (17.94)	3026 (16.88)	6376 (17.42)
Unemployed or retired	1358 (7.27)	1920 (10.71)	3278 (8.96)
Smokers, *n* (%)^∗^			
Never	11301 (60.51)	16931 (94.46)	28232 (77.14)
Former	1519 (8.13)	460 (2.57)	1979 (5.41)
Current	5856 (31.36)	533 (2.97)	6389 (17.46)
Drinkers, *n* (%)^∗^	1871 (10.02)	267 (1.49)	2138 (5.84)
Physical activity, *n* (%)			
Insufficient	12866 (68.89)	12323 (68.75)	25189 (68.82)
Sufficient	5810 (31.11)	5601 (31.25)	11411 (31.18)
Height (cm), mean (SD)^∗^	168.98 ± 6.25	162.85 ± 5.70	165.98 ± 6.73
Weight (cm), mean (SD)^∗^	65.53 ± 8.03	60.60 ± 7.13	63.12 ± 7.99
BMI (kg/m^2^), mean (SD)^#^	22.95 ± 2.54	22.88 ± 2.72	22.92 ± 2.63

Data are represented as the value (percentage) or mean ± SD. BMI: body mass index; SD: standard derivation. ^∗^*p* < 0.001 when comparing male with female. ^#^*p* < 0.05 when comparing male with female.

**Table 2:** Prevalence of overweight and obesity among various characteristics.

	Overweight		Obesity		Overweight/obesity	
	No. (%)	*P* value	No. (%)	*P* value	No. (%)	*P* value
Total	11248 (30.73)		1138 (3.11)		12386 (33.84)	
Gender		0.002		<0.001		0.152
Male	5874 (31.45)		511 (2.74)		6385 (34.19)	
Female	5374 (29.98)		627 (3.50)		6001 (33.48)	
Location		<0.001		0.117		<0.001
Urban	6139 (32.05)		569 (2.97)		6708 (35.03)	
Rural	5109 (29.28)		569 (3.26)		5678 (32.54)	
Age group (years)		<0.001		<0.001		<0.001
15-	1698 (22.18)		92 (1.20)		1790 (23.38)	
25-	1611 (27.68)		128 (2.20)		1739 (29.87)	
35-	2565 (32.35)		285 (3.59)		2850 (35.95)	
45-	2281 (34.44)		273 (4.12)		2554 (38.56)	
55-	1751 (35.73)		220 (4.49)		1971 (40.22)	
≥65	1342 (36.56)		140 (3.81)		1482 (40.37)	
Marriage		<0.001		<0.001		<0.001
Single	1737 (22.31)		101 (1.30)		1838 (23.61)	
Married or living with a partner	9100 (33.06)		999 (3.63)		10099 (36.61)	
Separated, divorced, or widowed	411 (31.84)		38 (2.94)		449 (36.47)	
Education		<0.001		<0.001		<0.001
Primary school and lower	3181 (32.65)		398 (4.08)		3579 (36.73)	
Middle school	6710 (30.34)		666 (3.01)		7376 (33.35)	
College and higher	1357 (28.64)		74 (1.56)		1431 (30.20)	
Occupation						
Manual	8427 (31.27)	<0.001	926 (3.44)	<0.001	9353 (34.71)	<0.001
Nonmanual	1656 (25.97)		93 (1.46)		1749 (27.43)	
Unemployed	1165 (35.54)		119 (3.63)		1284 (39.17)	
Smoker						
Never	8346 (29.56)	<0.001	874 (3.10)	0.033	9220 (32.66)	<0.001
Former	702 (35.47)		80 (4.04)		782 (39.51)	
Current	2200 (34.43)		184 (2.88)		2384 (37.31)	
Drinker		<0.001		0.062		<0.001
Yes	845 (39.52)		81 (3.79)		926 (43.31)	
No	10403 (30.19)		1057 (3.07)		11460 (33.25)	
Physical activity		<0.001		0.323		0.001
Insufficient	7892 (31.33)		768 (3.05)		8660 (34.38)	
Sufficient	3356 (29.41)		370 (3.24)		3726 (32.65)	
Sleep duration		<0.001		<0.001		<0.001
<6 h	295 (30.86)		56 (5.86)		351 (36.72)	
6-8 h	5308 (30.77)		500 (2.90)		5808 (33.67)	
8-10 h	5101 (30.23)		533 (3.16)		5634 (33.39)	
≥10	544 (35.86)		49 (3.23)		593 (39.09)	

Data are represented as value (percentage). Overweight: BMI 24-27.9 kg/m^2^; obesity: BMI ≥ 28 kg/m^2^; overweight/obesity: BMI ≥ 24 kg/m^2^.

**Table 3:** Factors associated with overweight/obesity by multivariate logistic regression analysis in Baoji adults.

	Overweight/obesity	
	OR (95% CI)	*P* value
Age (years)		
15-25	1	
25-34	1.167 (1.056~1.290)	<0.001
35-44	1.496 (1.350~1.658)	<0.001
45-54	1.697 (1.527~1.887)	<0.001
55-64	1.828 (1.634~2.045)	<0.001
≥65	1.820 (1.611~ .055)	<0.001
Location		
Urban	1	
Rural	0.904 (0.863~0.947)	<0.001
Marriage		
Single	1	
Married or living with a partner	1.266 (1.153~1.390)	<0.001
Separated, divorced, or widowed	1.130 (0.972~1.315)	0.113
Education		
Primary school and lower	1	
Middle school	1.039 (0.982~1.100)	0.186
College and higher	1.048 (0.955~1.149)	0.322
Occupation		
Manual	1	
Nonmanual	1.009 (0.935~1.089)	0.814
Unemployed or retired	1.183 (1.092~1.282)	<0.001
Smoker		
Never	1	
Former	1.116 (1.012~1.230)	0.027
Current	1.056 (0.989~1.129)	0.102
Drinker (vs. no)	1.410 (1.283~1.550)	<0.001
Physical activity		
Insufficient	1	
Sufficient	0.945 (0.899~0.993)	0.025
Sleep duration		
<6	1	
6-8 h	0.940 (0.820~1.079)	0.381
8-10 h	0.976 (0.851~1.120)	0.729
≥10 h	1.274 (1.075~1.510)	0.005

Data are represented as OR (95% CI). Overweight/obesity: BMI ≥ 24 kg/m^2^. OR was calculated with multivariable logistic regression analysis. OR: odds ratio; CI: confidence intervals.

## Data Availability

The original data used to support the findings of this study are available from the corresponding author upon request.
